# Whole Blood Viscosity and Its Associations with Age, Hematologic Indices, and Serum Biochemical Variables in Clinically Healthy Beagle Dogs and Korean Shorthair Cats

**DOI:** 10.3390/vetsci13010102

**Published:** 2026-01-20

**Authors:** Jinseok Son, Ji-Hyun Park, Seongjun Kim, Chae-Yeon Hong, Chang-Hwan Moon, Yong-ho Choe, Tae-sung Hwang, Jaemin Kim, Sung-Lim Lee, Dongbin Lee

**Affiliations:** 1 Institute of Animal Medicine, College of Veterinary Medicine, Gyeongsang National University, Jinju 52828, Republic of Korea; jinseok0327@naver.com (J.S.); 24qkr@naver.com (J.-H.P.); ch.moon@gnu.ac.kr (C.-H.M.); 2 Institute of Agriculture and Life Sciences, Gyeongsang National University, Jinju 52828, Republic of Korea; rlatjdwns991@naver.com (S.K.); jmkim85@gnu.ac.kr (J.K.); 3 College of Veterinary Medicine, Gyeongsang National University, Jinju 52828, Republic of Korea; xoojoox8@gmail.com (C.-Y.H.); yhchoe@gnu.ac.kr (Y.-h.C.); sllee@gnu.ac.kr (S.-L.L.); 4 Institute of Animal Medicine, Department of Veterinary Medical Imaging, College of Veterinary Medicine, Gyeongsang National University, Jinju 52828, Republic of Korea; hawngts@gnu.ac.kr

**Keywords:** whole blood viscosity, age association, hematologic variables, biochemical variables, dog, cat

## Abstract

This study investigated whether Whole blood viscosity (WBV) varies with age in clinically healthy Beagle dogs and Korean Shorthair cats and examined the hematologic and biochemical variables associated with WBV. WBV was measured across multiple shear rates using a scanning capillary viscometry; complete blood count (CBC) and serum chemistry profiles were also evaluated. Both species demonstrated characteristic shear-thinning behavior. WBV showed a strong association with red blood cell count (RBC), hematocrit (HCT), and hemoglobin (Hb) in both species, with additional association with serum proteins and cholesterol in dogs. No significant relationship between WBV and age was identified at any shear rate, and principal component analysis (PCA) revealed no age-related clustering in the viscosity profiles. These findings indicated that WBV does not exhibit meaningful age-dependent trends in healthy companion animals. This suggests that, in a clinical setting, deviations in normal WBV are more likely to influence underlying physiological or pathological factors than normal aging.

## Simple Summary

Whole blood viscosity (WBV) is a key determinant of blood flow, and age-related changes have been reported in humans. To determine whether similar patterns occur in companion animals, we measured WBV across multiple shear rates in healthy Beagle dogs and Korean Shorthair cats. We found that WBV did not vary with chronological age in either species, indicating that physiological aging alone did not alter blood viscosity in healthy animals. Instead, WBV was primarily associated with erythrocyte-related variables, with serum biochemical components providing an additional contribution in dogs. These findings suggest that WBV remains stable throughout adulthood in healthy dogs and cats, providing a useful context for interpreting viscosity measurements in veterinary practice.

## 1. Introduction

Whole blood viscosity (WBV) reflects the physical properties of blood that govern its flow through microvasculature, and, subsequently, determines how effectively oxygen is delivered to tissues [1,2]. Unlike Newtonian fluids, blood viscosity changes in response to the mechanical forces encountered during circulation. These changes result from the dynamic interaction between suspended erythrocytes and the surrounding plasma. Under low shear conditions, erythrocytes tend to form transient aggregates, increasing flow resistance. Under high shear, they elongate and align with the direction of flow, leading to a characteristic decrease in viscosity, a phenomenon known as shear thinning [1,3]. Variations such as hematocrit (HCT), plasma composition, and erythrocyte deformability influence these behaviors [1,2]. Because these factors collectively determine microvascular resistance, WBV serves as an integrated measure of the hemorheological state across a broad spectrum of physiological conditions.

Alterations in WBV have been documented in numerous human clinical studies, where elevated viscosity was associated with hypertension, metabolic dysregulation, and impaired renal function [4,5,6]. These associations demonstrate the sensitivity of WBV to pathophysiological states that alter erythrocyte deformability and plasma composition. While aging has also been reported to influence WBV, longitudinal research suggested that many observed changes are attributable to age-related comorbidities rather than to intrinsic aging processes [7,8,9,10]. This distinction is important because it underscores the need to determine whether WBV varies with chronological age in healthy individuals, independent of disease.

In veterinary medicine, WBV has been characterized in healthy dogs and cats, with reference values established via standardized scanning capillary viscometry [11,12]. Species-specific differences in hemorheological behavior have also been documented across mammals, highlighting how cellular and plasma characteristics influence viscosity profiles [13,14]. Despite this foundational knowledge, whether WBV changes with age in healthy companion animals remains unknown. This lack of age-specific data complicates the interpretation of WBV measurements in veterinary research and clinical contexts, as clinicians currently lack reference information to distinguish physiological variation associated with normal aging from WBV changes that are more suggestive of underlying systemic, metabolic, or hematologic disturbances. Previous veterinary studies reported baseline viscosity values without analyzing age-related trends, and most studies interpreted viscosity using single shear-rate measurements. From a clinical perspective, establishing whether WBV varies with normal aging is important for the interpretation of viscosity measurements in individual patients, as age-related reference information may help prevent misattribution of elevated WBV values to normal aging rather than potential pathological processes. Different shear rates reflect distinct hemorheological properties, from red blood cell count (RBC) aggregation at low shear to deformation-dependent flow at high shear. Ignoring shear-rate dependence may, therefore, lead to oversimplified or potentially misleading interpretations of blood flow behavior and microvascular resistance. Therefore, assessing WBV across multiple shear rates may provide a more comprehensive picture of potential age-related variation [13].

This study aimed to determine whether WBV varies with age in clinically healthy beagle dogs and Korean shorthair cats by measuring viscosity across a range of representative shear rates. A secondary objective was to examine associations between WBV and routine hematologic and biochemical variables to identify physiological factors of viscosity in healthy animals.

## 2. Materials and Methods

### 2.1. Animals and Study Population

Thirty-five clinically healthy beagle dogs and twenty-nine Korean Shorthair cats were prospectively enrolled. All animals underwent a physical examination, and their health status was confirmed via complete blood count (CBC) results and a standard 17-item serum chemistry panel; all values were required to fall within institutional reference ranges. Animals showing signs of dehydration, clinical abnormalities, or any medication administration within the preceding 30 days were excluded to minimize potential effects on WBV. Age, sex, and body weight distributions are presented in the Section 3 . Korean Shorthair cats were selected because they represent the most common domestic feline population in Korea, allowing the results to be broadly applicable to the general feline population. Beagle dogs were included as they are widely used as a standardized research breed with relatively homogeneous genetic and physiological characteristics, which facilitates consistency and comparison across studies. All procedures involving these dogs and cats were reviewed and approved by the Institutional Animal Care and Use Committee (IACUC) of Gyeongsang National University, Jinju, Republic of Korea (approval number: GNU-251219-T0273).

### 2.2. Blood Collection and Laboratory Analyses

CBC was analyzed using an automated hematology analyzer (ProCyte Dx^®^, IDEXX Laboratories, Westbrook, ME, USA). Measured variables included RBC, ×10^12^/L, hemoglobin concentration (HGB, g/dL), HCT (%), white blood cell (WBC, ×10^9^/L), and platelet count (PLT, ×10^9^/L). Standard erythrocyte and platelet indices, mean corpuscular volume (MCV, fL), mean corpuscular hemoglobin (MCH, pg), mean corpuscular hemoglobin concentration (MCHC, g/dL), red cell distribution width (RDW, %), and mean platelet volume (MPV, fL)were also obtained.

Serum biochemical analyses were performed using an automated chemistry analyzer (Catalyst One^®^, IDEXX Laboratories, Westbrook, ME, USA). Quantified parameters comprised albumin (ALB, g/dL), total protein (TP, g/dL), globulin (GLOB, g/dL), cholesterol (CHOL, mg/dL), glucose (GLU, mg/dL), blood urea nitrogen (BUN, mg/dL), creatinine (CRE, mg/dL), alanine aminotransferase (ALT, U/L), alkaline phosphatase (ALKP, U/L), γ-glutamyl transferase (GGT, U/L), amylase (AMYL, U/L), lipase (LIPA, U/L), calcium (Ca, mg/dL), phosphorus (PHOS, mg/dL), and total bilirubin (TBIL, mg/dL). All blood samples were collected under standardized conditions. Animals were fasted for at least 8 h prior to blood collection, allowed to rest in a calm environment, and samples were obtained during daytime hours to minimize potential circadian and activity-related variability.

### 2.3. Whole Blood Viscosity Measurement

WBV was measured using a Casson U-shaped scanning capillary tube viscometer (SCTV; Rheovis-01; Biorheologics Co., Ltd., Jeonju, Republic of Korea). This device measures viscosity by monitoring blood flow via a precision capillary tube under controlled pressure conditions. All measurements were conducted at 37 °C to maintain physiological temperature. For each sample, WBV was recorded across shear rates ranging from 1 to 1000 s^−1^. For subsequent statistical analysis, WBV values measured at 1 and 300 s^−1^ were designated as representing diastolic and systolic WBV, respectively.

### 2.4. Principal Component and Statistical Analyses

Principal component analysis (PCA) was applied to WBV values measured at seven shear rates (1, 5, 10, 50, 100, 150, and 300 s^−1^). This analysis served to summarize overall viscosity profiles and to evaluate potential age-related clustering in the data. Prior to PCA, WBV variables measured at different shear rates were standardized to account for differences in variance across shear rates. Principal components were retained based on eigenvalues greater than 1 and cumulative explained variance. Associations between WBV (at 1 and 300 s^−1^) and individual hematologic or biochemical variables were examined using Pearson correlation coefficients. WBV values at 1 and 300 s^−1^ were selected for correlation analyses because these shear rates represent low-shear (diastolic) and high-shear (systolic) hemorheological conditions, respectively, and are commonly used to reflect clinically relevant extremes of blood flow behavior. Statistical significance was defined as *p* < 0.05. All analyses were performed using R software (version 4.3.2; R Foundation for Statistical Computing, Vienna, Austria).

## 3. Results

A total of 35 Beagle dogs and 29 Korean shorthair cats were included in the final analyses. The Beagle group consisted of 17 males and 18 females, with body weights ranging from 6.0 to 11.0 kg, and ages from 0.6 to 14 years. The Korean Shorthair group comprised 16 males and 13 females, with body weights ranging from 3.0 to 7.4 kg, and ages from 0.6 to 9 years. All animals had CBC and serum chemistry values within institutional reference intervals, confirming their clinical health before WBV measurement. Detailed characteristics and laboratory data of the study population are provided in Supplementary Tables S1 and S2.

### 3.1. Principal Component Analysis of WBV by Age

**Figure 1:**
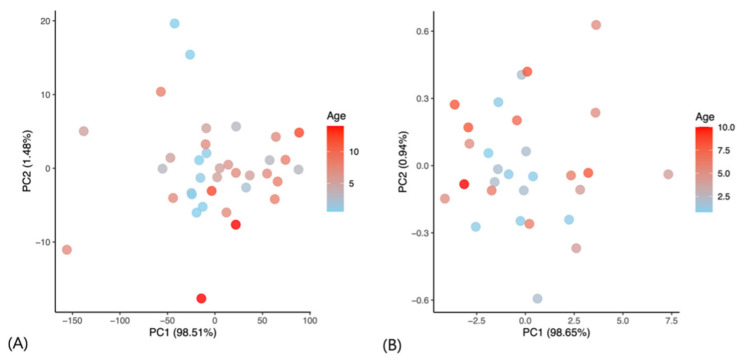
Principal component analysis (PCA) score plots of whole blood viscosity (WBV) in Beagle dogs (**A**) and Korean shorthair cats (**B**). PC1, first principal component; PC2, second principal component.

PCA of WBV values across seven shear rates (1–300 s^−1^) was performed separately for Beagle dogs and Korean Shorthair cats. In Beagle dogs (Figure 1A), the first principal component (PC1) and the second principal component (PC2) explained 98.51% and 1.48% of the total variance, respectively. The PCA score plot revealed no clustering or age gradient. In Korean Shorthair cats (Figure 1B), PC1 and PC2 accounted for 98.65% and 0.94% of the variance, respectively, with no age-related grouping in the score plot. WBV profiles showed no significant association with chronological age in both beagle dogs and Korean Shorthair cats across the studied age ranges.

### 3.2. Associations Between WBV and CBC Variables

**Figure 2:**
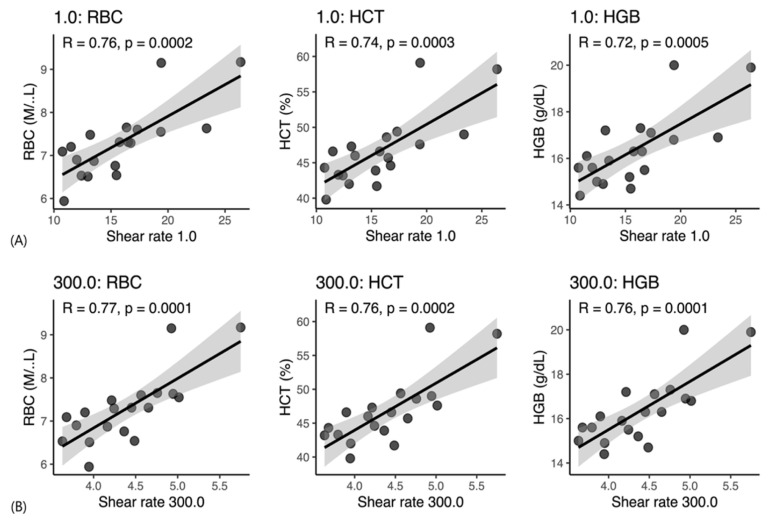
Scatterplots showing correlations between whole blood viscosity (WBV) and hematological parameters in Beagle dogs. (**A**) Low shear rate (1 s^−1^), representing diastolic blood flow conditions; (**B**) High shear rate (300 s^−1^), representing systolic blood flow conditions. RBC, red blood cell count; HCT, hematocrit; HGB, hemoglobin; R, Pearson’s correlation coefficient; *p*, *p*-value.

In Beagle dogs, WBV showed strong positive correlations with erythrocyte-related indices at both 1 s^−1^ and 300 s^−1^ (Figure 2A,B). Significant positive correlations were observed between WBV and RBC count, HCT, and Hb (r = 0.72–0.77, *p* ≤ 0.0005), confirming erythrocyte mass as the primary hematologic determinant of viscosity. In contrast, leukocyte-related parameters showed no significant correlations with WBV; both WBC count and neutrophil percentage demonstrated weak, non-significant correlations (*p* > 0.30).

**Figure 3:**
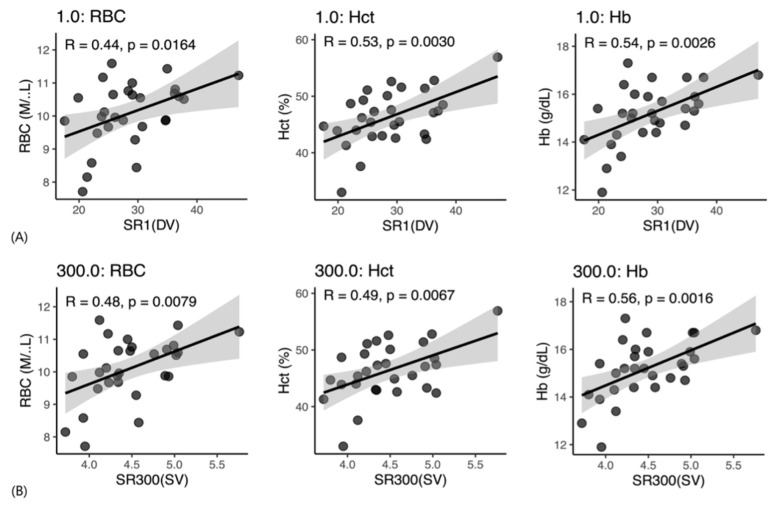
Scatterplots showing correlations between whole blood viscosity (WBV) and hematological parameters in Korean shorthair cats. (**A**) Low shear rate (1 s^−1^), representing diastolic blood flow conditions; (**B**) High shear rate (300 s^−1^), representing systolic blood flow conditions. RBC, red blood cell count; HCT, hematocrit; HGB, hemoglobin; R, Pearson’s correlation coefficient; *p*, *p*-value.

Korean short-air cats exhibited a comparable pattern (Figure 3A,B). WBV correlated positively with RBC count, HCT, and Hb (r = 0.44–0.56, *p* ≤ 0.016), while leukocyte variables, including WBC count and neutrophil percentage, showed negligible associations with WBV (*p* > 0.70).

### 3.3. Associations Between WBV and Serum Chemistry Variables

**Figure 4:**
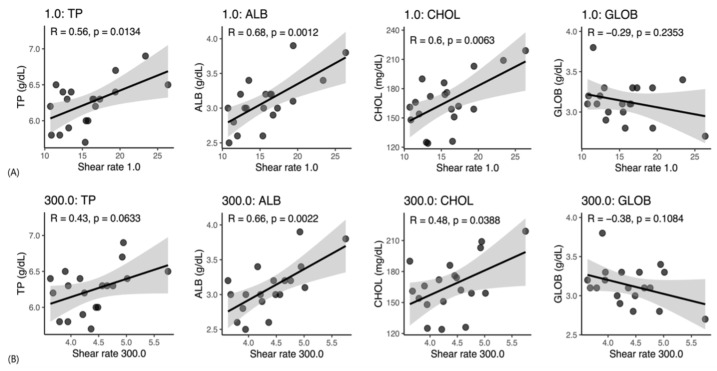
Scatterplots showing correlations between whole blood viscosity (WBV) and serum biochemical parameters in Beagle dogs. (**A**) Low shear rate (1 s^−1^), representing diastolic blood flow conditions; (**B**) High shear rate (300 s^−1^), representing systolic blood flow conditions. TP, total protein; ALB, albumin; GLOB, globulin; CHOL, cholesterol; R, Pearson’s correlation coefficient; *p*, *p*-value.

In Beagle dogs, WBV showed measurable associations with all four serum biochemical variables (Figure 4A,B). At a shear rate of 1 s^−1^, WBV demonstrated significant positive correlations with total protein, ALB, and CHOL (r = 0.56, 0.68, and 0.60, respectively; all *p* ≤ 0.0134), while globulin showed a non-significant negative correlation (r = −0.29, *p* = 0.2353). At 300 s^−1^, significant correlations persisted for ALB and CHOL (r = 0.66 and 0.48, respectively; *p* ≤ 0.0388), with total protein showing a positive trend (r = 0.43, *p* = 0.0633) and globulin also demonstrating a non-significant negative association (r = −0.38, *p* = 0.1084).

**Figure 5:**
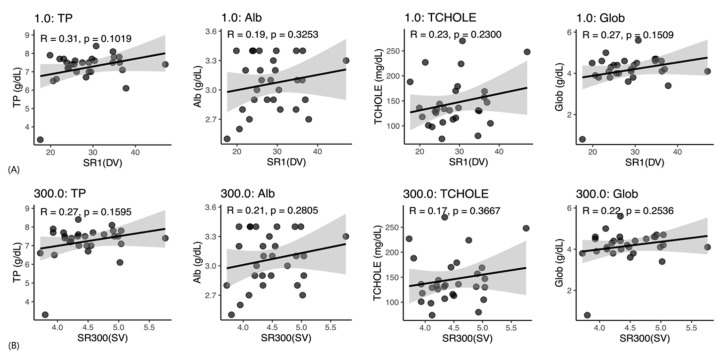
Scatterplots showing correlations between whole blood viscosity (WBV) and serum biochemical parameters in Korean shorthair cats. (**A**) Low shear rate (1 s^−1^), representing diastolic blood flow conditions; (**B**) High shear rate (300 s^−1^), representing systolic blood flow conditions. TP, total protein; ALB, albumin; GLOB, globulin; CHOL, cholesterol; R, Pearson’s correlation coefficient; *p*, *p*-value.

In Korean Shorthair cats (Figure 5A,B), none of the four serum chemistry variables were significantly associated with WBV. Serum biochemical variables, including ALB, globulin, total protein, and CHOL, showed weak correlations with WBV (r = 0.17–0.31), none of which were statistically significant (*p* > 0.10).

## 4. Discussion

This study investigated the association between WBV and age in clinically healthy dogs and cats and assessed the hematologic and biochemical factors contributing to viscosity across physiological shear conditions. When WBV was evaluated both at individual shear rates and as integrated, shear-dependent profiles via PCA, no evidence of age-related variations was identified in either species. This combined analytical approach allowed for the assessment of both discrete rheological measurements and overall viscosity behavior across a range of flow environments relevant to physiological circulation, thereby reducing reliance on isolated shear-specific observations. Viscosity is most strongly associated with erythrocyte-related variables, RBC count, HCT, and Hb concentration, reflecting the central rheologic influence of red cell mass, as described in fundamental hemorheology [1,2,15]. The consistency of these associations across both low- and high-shear rates underscores the pivotal role of erythrocyte characteristics in determining WBV, independent of flow conditions. This finding aligns with previous interspecies comparisons demonstrating a strong dependence of WBV on cellular rheological factors [3,13] and supports the concept that erythrocyte-related parameters are primary determinants of whole-blood flow properties under healthy conditions.

Although human clinical studies have reported age-associated increases in WBV, more recent longitudinal research suggested these changes primarily reflect comorbidities, such as chronic low-grade inflammation, metabolic dysregulation, oxidative stress, and vascular remodeling, rather than chronological aging itself [7,8,9,10]. These comorbidity-driven influences include increased fibrinogen, altered plasma protein composition, and impaired erythrocyte deformability, all of which elevated viscosity through distinct rheologic pathways [1,2,16]. In our study, the exclusion of animals with clinical or biochemical abnormalities potentially minimized these confounders and allowed for the evaluation of WBV under conditions more representative of physiological health. This design feature reduced the likelihood that subclinical disease processes contributed to the observed rheological profiles. The absence of age-related clustering in the PCA, which evaluated integrated viscosity profiles rather than single measurements, supports the conclusion that WBV remains physiologically stable throughout adulthood in healthy companion animals. By incorporating viscosity data across multiple shear conditions, this approach strengthens confidence that the observed findings are not driven by isolated shear-dependent effects. This finding contrasts with earlier assumptions based on human data; however, it conceptually aligns with recent evidence suggesting that viscosity abnormalities should be interpreted within the broader context of systemic health rather than attributed to age alone. Recent studies in dogs have also documented age-related alterations in immune and metabolic biomarkers without directly assessing WBV, suggesting that rheological stability may coexist with other physiological aging processes in this species [17].

The strong correlations observed between WBV and erythrocyte indices align with the biomechanical principles describing how RBCs regulate blood flow under varying shear environments. At low shear rates, reversible erythrocyte aggregation increases flow resistance, a process highly sensitive to HCT and RBC surface properties [2,3]. At high shear rates, RBC deformability becomes the primary determinant of viscosity, although HCT remains a substantial influence by modulating cell–cell interactions and suspension density [1,2,15]. These shear-dependent mechanisms account for the robust correlations observed between WBV and RBC count, HCT, and HGB across the range of shear conditions examined in this study. Together, these findings illustrate how erythrocyte concentration and mechanical behavior interact to influence blood flow under physiologically relevant conditions, reflecting the dynamic balance between cellular interactions and hydrodynamic forces. Importantly, these relationships were consistently observed across the tested shear spectrum, suggesting that erythrocyte-driven effects on viscosity persist under diverse flow states encountered in vivo. Furthermore, interspecies differences in erythrocyte morphology, including cell diameter, surface-area-to-volume ratio, and membrane stiffness, are known to influence aggregation and deformability, thereby contributing to species-specific viscosity behaviors [13,14]. Our findings reinforce this concept by demonstrating similar erythrocyte-driven determinants in both species, with distinct species-specific responses to plasma biochemical factors.

Notable species differences emerged in the contribution of plasma biochemical components to WBV. In cats, serum protein and CHOL levels showed no significant association with viscosity at either shear rate. This finding aligns with the established understanding that feline erythrocytes exhibit minimal rouleaux formation and intrinsically weak aggregation tendencies compared with canine erythrocytes [13,14]. Since plasma proteins primarily influence WBV by prompting aggregation and increasing plasma viscosity, the low aggregability of feline RBCs may reduce the physiological detectability of plasma phase effects. In other words, modest variation in the suspending phase may have a limited impact on measured WBV when erythrocyte aggregation is inherently constrained in practice. This interpretation is consistent with the concept that, under healthy conditions, cellular factors may dominate the rheological signal in cats, particularly at low shear, where aggregation-dependent effects would otherwise be expected. In addition, the limited responsiveness of feline erythrocytes to plasma-mediated interactions may further dampen measurable viscosity changes across physiological conditions. Furthermore, the relatively narrow biochemical range observed in healthy cats may also limit the strength of measurable correlations, thereby reducing statistical power to detect small plasma-driven contributions within a physiologic interval. Taken together, these factors suggest that plasma-related influences on WBV in cats may become more apparent only under pathological conditions that substantially alter plasma composition or erythrocyte behavior.

In contrast, WBV in dogs was significantly associated with ALB, total protein, and CHOL levels. ALB and other plasma proteins contributed directly to plasma viscosity and modulate erythrocyte aggregation through physicochemical interactions [16]. Elevated plasma protein concentration increases the suspension phase viscosity and promotes bridging or depletion interactions that facilitate erythrocyte aggregation under low-shear conditions. These effects enhance cell–cell interactions and increase flow resistance, particularly in shear environments where aggregation-dependent mechanisms predominate. Such plasma-mediated influences may amplify viscosity changes even when erythrocyte indices remain within physiological ranges. CHOL influenced erythrocyte membrane fluidity and could impair deformability, thereby elevating viscosity under higher shear conditions [18]. Alterations in membrane lipid composition may affect cytoskeletal dynamics and mechanical flexibility of erythrocytes, further contributing to shear-dependent viscosity changes. Similar observations have been reported in previous studies evaluating WBV in Beagle dogs, where both erythrocyte mass and plasma constituents contributed to viscosity variability [11]. Therefore, our findings align with the established model of combined cellular- and plasma-phase determinants of WBV in canine hemorheology and support species-specific interpretation of plasma-related effects.

This study provided important insights for the clinical interpretation of WBV in veterinary medicine. In humans, elevated WBV has been linked to impaired microcirculatory perfusion, reduced oxygen delivery efficiency, endothelial dysfunction, and a higher risk of cerebrovascular and cardiovascular events [4,15,19,20,21]. These findings underscore the role of WBV as an integrative indicator of circulatory and metabolic status. Because WBV remains stable with age in healthy dogs and cats, elevated viscosity in clinical patients may more reliably indicate underlying metabolic, inflammatory, or hemodynamic disturbances rather than age-related change. In this context, WBV assessment may assist clinicians in distinguishing pathological alterations from physiological aging, particularly in older animals in which multiple subclinical processes may coexist. Such differentiation is often challenging in geriatric patients when age-related changes overlap with early disease manifestations and clinical signs are nonspecific. Interpretation of WBV alongside routine hematologic and biochemical parameters may therefore enhance overall clinical assessment and support more informed diagnostic decision-making. In addition, serial evaluation of WBV may provide additional information when monitoring disease progression or response to therapy. Existing veterinary reference values for WBV, established using comparable viscometric methods, can serve as robust baselines for identifying clinically relevant abnormalities [11,12]. As the focus on hemodynamic biomarkers grows in veterinary practice, WBV represents a valuable adjunct parameter for assessing systemic diseases, stratifying risk, and monitoring therapeutic responses in conjunction with conventional diagnostic indices over time.

This study has several limitations. The use of a single breed for each species reduced variability; however, this limited generalizability across broader canine and feline populations. Blood rheology varies with breed, erythrocyte morphology, and metabolic profiles [13,14], suggesting the need for expanded studies that include multiple breeds representing a wider range of hematologic characteristics. The sample size was modest, and the cross-sectional design precluded longitudinal assessment of individual animals over their lifespan, limiting evaluation of intra-individual changes over time. Additionally, several key rheological parameters, including plasma viscosity, fibrinogen concentration, erythrocyte deformability indices, and aggregation kinetics, were not measured. These parameters are major determinants of viscosity and would offer deeper mechanistic insights into species differences [1,2,3]. The absence of direct measurements restricts interpretation of the relative contributions of plasma- and cell-based factors and limits mechanistic inference. Future research should incorporate these variables, broaden the demographic range, and include animals with naturally occurring diseases, such as chronic kidney disease, endocrine disorders, systemic inflammation, and cardiovascular disease. Such studies would clarify the diagnostic and prognostic utility of WBV in veterinary medicine and determine whether disease-specific viscosity profiles can be defined more clearly.

In summary, these results enhance our understanding of the physiological determinants of WBV in healthy dogs and cats. They illustrate how erythrocyte mechanics, plasma composition, and species-specific rheologic traits collectively govern whole-blood flow behavior under physiological conditions. By clarifying the relative contributions of cellular and plasma-related factors, the present findings help refine the interpretation of viscosity measurements in clinically normal animals and provide context for interindividual variability observed in practice. These findings provide a necessary foundation for future research, aiming to integrate blood viscosity assessment into veterinary diagnostic frameworks and to identify pathological processes that disrupt rheological homeostasis. Such efforts may ultimately support the development of more refined diagnostic and monitoring strategies for systemic disease in veterinary medicine and contribute to improved clinical decision-making.

## 5. Conclusions

This study demonstrated that WBV remains stable throughout adulthood in clinically healthy beagle dogs and Korean Shorthair cats. WBV was most strongly influenced by erythrocyte-related variables, including RBC count, HCT, and Hb, while plasma components contributed to WBV in dogs but not in cats. No age-related changes were detected through either shear-specific analysis or PCA of integrated viscosity profiles, indicating that chronological age alone does not influence WBV under healthy physiological conditions. These consistent findings across multiple analytical approaches reinforce the conclusion that WBV is tightly regulated within a physiological range in the absence of overt disease. These results provide an important context for the clinical interpretation of WBV, suggesting that elevated viscosity in veterinary patients is more likely to reflect underlying systemic or metabolic disturbances than normal aging. This distinction is particularly relevant in clinical settings where age-related physiological variation may otherwise complicate interpretation. The results establish a physiological baseline for future research into disease-associated alterations in WBV, enabling clearer differentiation between health- and disease-related rheological changes. Moreover, these findings support the broader integration of viscometric assessments into veterinary diagnostic and research frameworks by emphasizing their potential value in identifying clinically meaningful deviations from normal rheological homeostasis.

## Data Availability

The original contributions presented in this study are included in the article/Supplementary Materials. Further inquiries can be directed to the corresponding author.

## Supplementary Materials

The following supporting information can be downloaded at https://www.mdpi.com/article/10.3390/vetsci13010102/s1. Table S1 presents the characteristics, complete blood count, and serum biochemical variables in clinically healthy Beagle dogs. Table S2 presents the characteristics, complete blood count, and serum biochemical variables in clinically healthy Korean Shorthair cats.

## Abbreviations

The following abbreviations are used in this manuscript:

ALB	Albumin
ALKP	Alkaline phosphatase
ALT	Alanine aminotransferase
AMYL	Amylase
BUN	Blood urea nitrogen
Ca	Calcium
CBC	Complete blood count
CHOL	Cholesterol
CRE	Creatinine
GGT	γ-glutamyl transferase
GLU	Glucose
GLOB	Globulin
GNU	Gyeongsang National University
Hb/HGB	Hemoglobin
HCT	Hematocrit
IACUC	Institutional Animal Care and Use Committee
LIPA	Lipase
MCH	Mean corpuscular hemoglobin
MCHC	Mean corpuscular hemoglobin concentration
MCV	Mean corpuscular volume
MPV	Mean platelet volume
PCA	Principal component analysis
PC1	First principal component
PC2	Second principal component
PHOS	Phosphorus
PLT	Platelet count
RBC	Red blood cell count
RDW	Red cell distribution width
s^−1^	Per second (shear rate unit)
TBIL	Total bilirubin
TP	Total protein
WBV	Whole blood viscosity
WBC	White blood cell count
