# Supplementary material for: Whole Blood Viscosity and Its Associations with Age, Hematologic Indices, and Serum Biochemical Variables in Clinically Healthy Beagle Dogs and Korean Shorthair Cats
Source: Vet Sci. 2026 Jan 20;13(1):102. doi: 10.3390/vetsci13010102 (PMC12846588; doi:10.3390/vetsci13010102)
Supplement: Supplementary file 1 [file vetsci-13-00102-s001.zip › vetsci-4109156-supplementary.pdf]

Table S1. Baseline characteristics, complete blood count, and serum biochemical variables in clinically healthy Beagle dogs (n = 35). RBC, red blood cell count; HCT, hematocrit; HGB, hemoglobin; WBC, white blood cell count; PLT, platelet count; SD, standard deviation.

Parameter	Unit	Mean ± SD	Reference interval
Age	years	6.4 ± 3.2	–
Sex (male/female)	n	17 / 18	–
Body weight	kg	9.2 ± 1.5	–
RBC	$\times 10^6/\mu L$	6.85 ± 1.16	5.5 – 8.5
HCT	%	43.7 ± 7.8	37 – 55
HGB	g/dL	15.3 ± 2.6	12 – 18
WBC	$\times 10^3/\mu L$	7.76 ± 2.40	6.0 – 17.0
PLT	$\times 10^3/\mu L$	321.5 ± 124.9	200 – 500
Total protein	g/dL	6.33 ± 1.11	5.4 – 7.8
Albumin	g/dL	3.07 ± 0.60	2.6 – 4.0
Globulin	g/dL	4.14 ± 6.06	2.5 – 4.5
Cholesterol	mg/dL	181.1 ± 88.8	110 – 320

Table S2. Baseline characteristics, complete blood count, and serum biochemical variables in clinically healthy Korean Shorthair cats (n = 29). RBC, red blood cell count; HCT, hematocrit; HGB, hemoglobin; WBC, white blood cell count; PLT, platelet count; SD, standard deviation.

Parameter	Unit	Mean ± SD	Reference interval
Age	years	4.06 ± 2.76	–
Sex (male/female)	n	16 / 13	–
Body weight	kg	5.23 ± 1.33	–
RBC	$\times 10^6/\mu L$	10.08 ± 1.05	5.0 – 10.0
HCT	%	46.8 ± 6.0	30 – 45
HGB	g/dL	15.2 ± 1.3	8.0 – 15.0
WBC	$\times 10^3/\mu L$	8.79 ± 2.77	5.5 – 19.5
PLT	$\times 10^3/\mu L$	216.7 ± 94.6	300 – 800
Total protein	g/dL	7.29 ± 0.63	5.7 – 7.8
Albumin	g/dL	3.02 ± 0.29	2.5 – 3.9
Globulin	g/dL	4.27 ± 0.86	2.8 – 5.1
Cholesterol	mg/dL	145.3 ± 57.8	70 – 220
